# Untargeted Metabolomics Reveals Distinct Soil Metabolic Profiles Across Land Management Practices

**DOI:** 10.3390/metabo15120783

**Published:** 2025-12-04

**Authors:** Zane A. Vickery, Hector F. Castro, Stephen P. Dearth, Eric D. Tague, Aimée T. Classen, Jessica A. Moore, Michael S. Strickland, Shawn R. Campagna

**Affiliations:** 1 Department of Chemistry, University of Tennessee, Knoxville, TN 37996, USA; zvickery@vols.utk.edu (Z.A.V.); eric.tague@thermofisher.com (E.D.T.); 2 UT Biological and Small Molecule Mass Spectrometry Core, Knoxville, TN 37996, USA; 3 Department of Ecology and Evolutionary Biology, University of Michigan, Ann Arbor, MI 48109, USA; aclassen@umich.edu; 4 Department of Microbiology, University of Tennessee, Knoxville, TN 37996, USA; 5 Department of Soil and Water Systems, University of Idaho, Moscow, ID 83844, USA; mstrickland@uidaho.edu

**Keywords:** untargeted metabolomics, soil metabolome, land management practices, UHPLC-HRMS, biogeochemical cycling

## Abstract

**Background/Objectives:** Land management practices strongly influence soil biochemical processes, yet conventional soil measurements often overlook dynamic small-molecule variation underlying nutrient cycling and microbial activity. This study aimed to evaluate whether MS^1^-based untargeted metabolomics can resolve meaningful biochemical differences among soil systems under distinct land management practices. **Methods:** Soils from six land-use types—conventional cultivation, organic cultivation, pasture, white pine, tulip poplar, and hardwood forest—were analyzed using ultra-high-performance liquid chromatography coupled with high-resolution mass spectrometry (UHPLC-HRMS). Multivariate analyses, including PLS-DA, were performed to evaluate metabolic variation across systems. Both identified metabolites and unknown spectral features (MSI Level 4) were assessed, and biosynthetic class assignment of unknown features was performed using NPClassifier. **Results:** Metabolic features revealed clear separation between land management systems, demonstrating distinct chemical fingerprints across ecosystems. While conventional elemental ratios (e.g., C/N) showed minimal differentiation, phosphorus-related stoichiometric ratios (C/P and N/P) displayed strong land-use-dependent differences. NPClassifier superclasses highlighted unique chemical patterns, with forest soils enriched in diverse secondary metabolites, cultivated soils characterized by simplified profiles, and pasture soils dominated by microbial membrane lipids and alkaloids. **Conclusions:** Untargeted MS^1^-based metabolomics effectively distinguished soil systems under different land-use practices and revealed ecologically meaningful variation even without complete structural identification. This study demonstrates that an MS^1^-only workflow leveraging unknown spectral features can robustly distinguish soil systems, underscoring their value in untargeted metabolomics analyses.

## 1. Introduction

Land management practices play an important role in shaping the health and functionality of soil ecosystems, influencing nutrient cycling, soil structure, and microbial activity. Conventional management often involves synthetic fertilizers, pesticides, herbicides, mechanical tillage, and irrigation. In contrast, organic management restricts synthetic inputs, relying on compost, crop rotation, biological pest control, and other sustainable techniques to support soil fertility and ecological stability [1]. Forest soils, which are shaped by natural plant detritus and minimal disturbance, accumulate organic matter through slower decomposition processes. In fact, soils under hardwood and coniferous forests differ in their chemical and biological profiles, with variations in pH, nutrient availability, and microbial community composition [2]. These variations affect nutrient cycling and organic matter turnover, ultimately influencing soil function.

Beyond individual ecosystems, land management practices broadly impact biogeochemical processes, including organic matter decomposition, nutrient cycling, and soil structure [3]. Studies find that different land-use types significantly alter nutrient availability and organic matter dynamics, with downstream effects on soil fertility and ecosystem function [4]. Similarly, agricultural management practices can modify soil characteristics and reshape biogeochemical cycles [5]. Together, these findings highlight the central role of land management in controlling soil chemistry, nutrient dynamics, and broader ecosystem processes, providing a foundation for exploring soil biochemical variation through metabolomic approaches.

Traditional soil analyses generally rely on physical and chemical measurements, such as bulk elemental composition, soil texture, and microbial community composition, to assess soil health and nutrient pools [6]. While these approaches provide valuable environmental information, they primarily capture broader chemical trends and may overlook dynamic biochemical processes occurring over shorter time periods and at smaller molecular scales [7]. In contrast, metabolomics offers a more detailed view of soil biochemistry by profiling the diverse small molecules involved in nutrient cycling, organic matter turnover, and chemical interactions within the soil matrix [8,9,10,11,12,13]. This approach has been increasingly applied to soils, sediments, and rhizosphere systems. An untargeted metabolomics approach can reveal both known and novel metabolites associated with microbial activity, plant inputs, and environmental perturbations.

However, soils present challenges for metabolomic analysis. As complex systems composed of organic and inorganic material, diverse chemical inputs, and intricate biogeochemical interactions, soils generate highly complex metabolite profiles. These features make it difficult to fully characterize soil biochemistry using traditional targeted metabolomics approaches, which often focus on limited sets of known compounds. As a result, untargeted methods that capture broad metabolomic variation are essential for uncovering the biochemical diversity within soils.

Untargeted metabolomics enables the simultaneous detection of hundreds to thousands of metabolites spanning a wide array of biochemical pathways. However, even with these advancements, only a small fraction of detected metabolites (less than 5%) can be confidently identified, leaving most of the soil metabolome uncharacterized [14]. This largely unexplored fraction, often referred to as the “dark metabolome,” holds significant potential for revealing novel biogeochemical patterns and environmental processes [15]. These untapped chemical signatures highlight the importance of continued exploration of soil biochemical complexity beyond known compounds.

For this study, ultra-high-performance liquid chromatography coupled with high-resolution mass spectrometry (UHPLC-HRMS)-based metabolomics was employed to examine the soil metabolomes of six distinct land management practices: conventional cultivation (CC), organic cultivation (OC), hardwood forest (HF), white pine (WP), tulip poplar (TP), and pastureland. Each management type differentially influences soil properties, potentially resulting in shifts in metabolite composition, particularly among compounds related to nutrient cycling and organic matter turnover.

Pasture soils were selected as a reference baseline due to their minimal anthropogenic disturbance and relatively stable management history. Forest soils, which are also largely undisturbed, differ in that they develop primarily through plant detritus decomposition and closed nutrient cycling, without any active management. In contrast, cultivated soils represent highly managed systems, undergoing regular fertilizer input, mechanical tillage, and other disturbances that can dramatically alter nutrient availability and soil structure. These contrasts in land management and disturbance intensity were expected to drive differences in soil biochemical profiles.

It was hypothesized that significant changes in elemental ratios and metabolite composition would be observed across land management practices, providing insight into their impacts on soil biogeochemistry. Untargeted metabolomics was applied to identify distinct metabolic profiles associated with each land management practice and to broadly classify metabolite groups contributing to variation across systems. By characterizing these soil metabolomes, this research aims to advance understanding of the biogeochemical processes underlying soil health and ecosystem function.

## 2. Materials and Methods

Field soils were collected from six land cover or management types representative of the Ridge and Valley Province of Virginia, USA: conventional cultivation (i.e., inorganic fertilizer and pesticide inputs, typically under corn–soybean rotation), organic cultivation (mixed vegetables), mixed deciduous forest (primarily *Quercus* sp. and *Fagus grandifolia*), tulip poplar (*Liriodendron tulipifera*) stands, white pine (*Pinus strobus*) stands, and pastureland (primarily *Festuca arundinacea*).

Coordinates for each sampling location were as follows:

− Conventional cultivation: N37° 11.9′, W80° 34.5′
− Organic cultivation: N37° 15.5′, W80° 35.8′
− Deciduous forest: N37° 15.4′, W80° 35.8′
− Tulip poplar stand: N37° 15.3′, W80° 35.8′
− White pine stand: N37° 11.8′, W80° 35.0′
− Pasture: N37° 12.1′, W80° 34.0′

For each experimental group, four random soil cores (10 cm depth) were collected and then sieved (4 mm) and homogenized. Samples from each group were pooled and homogenized in the field. Subsamples were immediately frozen using liquid nitrogen and stored at −80 °C until metabolomic analysis. Before metabolomic analysis, subsamples were pulverized and homogenized three times using a mortar and pestle under liquid nitrogen. Grinding, weighing, and extraction of metabolites were carried out inside a cold room at 4 °C.

The soils from this study were collected from a diverse set of land management types (cropland, pasture, and forest) within the Ridge and Valley Province of Virginia. These sites reflect the region’s typical ecological and soil conditions and have been the subject of several prior microbial and biogeochemical studies. For example, a study conducted soil health assessments at a site in the Blacksburg area, within the same regional landscape as our study, representing long-term no-till corn systems with cover crops [16]. A nearby pasture site was also used to investigate how volatile organic compounds from decomposing litter influence soil microbial communities and carbon cycling [17]. In another study, microbial characteristics were assessed across actively managed farms throughout Virginia. Those farms included sites with fescue pastures and corn/soybean rotations on soils similar to those in this dataset [18]. This highlights the regional relevance of the sampling locations in this study.

### 2.1. Soil Metabolite Extraction

The acidic acetonitrile extraction introduced by Rabinowitz and Kimball was designed as a holistic method and validated across diverse microorganisms, which are metabolically complex systems [19]. Since its introduction, the approach has been widely adopted beyond mammalian applications and is now established across multiple biological and environmental matrices. It has been applied to soil systems, marine and aquatic environments, and environmental microbial consortia [20,21,22]. The same solvent system is also described in metabolomics best-practice reviews and adapted for plant tissues [23,24]. These prior validations support its suitability for extracting water-soluble metabolites from complex soil matrices.

The homogenized soil samples (*n* = 4) were weighed into 30 mg aliquots and analyzed at the Biological and Small Molecules Mass Spectrometry Core at the University of Tennessee, Knoxville, TN (RRID: SCR 021368). The experiment utilized a previously established untargeted metabolomics procedure for the extraction and analysis of water soluble metabolites [19]. Ultra-high performance liquid chromatography grade solvents (Fisher Scientific, Hampton, NH, USA) were used during the entirety of the experiment. The extraction solvent, composed of methanol, acetonitrile, and water with formic acid at a final concentration of 0.1 M in a 2:2:1 ratio, was added to each sample at a volume of 1.3 mL in a cold room at 4 °C.

Once mixed, the samples were shaken on an orbital platform shaker (Bellco, Vineland, NJ, USA) at 4 °C for 20 min. The samples were centrifuged at 15,000 rpm for 5 min before collection of the supernatant. Additionally, 200 µL of extraction solvent was added to the remaining pellet, and this mixture was shaken for an additional 20 min at 4 °C. Then, centrifugation for five minutes at 15,000 rpm occurred prior to collection of supernatants. Both supernatants were then combined and allowed to evaporate under high purity nitrogen. Once dried, 300 µL of water was added to resuspend the samples prior to mass spectral analyses. Samples were immediately placed in autosampler trays at 4 °C for mass spectrometric analysis.

### 2.2. UPHLC-HRMS Metabolomics Analysis

The analysis was conducted using an ultra-high-performance liquid chromatography system coupled with a high resolution Orbitrap mass spectrometer (UHPLC-HRMS), and a previously established metabolomics analysis method [25]. In short, the chromatographic separation employed a reverse-phase 25-min water:methanol gradient solvent system with tributylamine (TBA) used as an ion pairing reagent. Resuspended samples were stored at 4 °C during analysis and the injection volume was 10 µL. The water-soluble metabolites were separated on a Synergi 2.5 micron reverse-phase Hydro-RP 100 Å, 100 × 2.00 mm LC column (Phenomenex, Torrance, CA, USA) kept at 25 °C and using an UltiMate 3000 pump (Dionex, Sunnyvale, CA, USA). The eluent was introduced into the MS via an electrospray ionization source (ESI) coupled to an Exactive™ Plus Orbitrap Mass Spectrometer (Thermo Scientific, Waltham, MA, USA) through a 0.1 mm internal diameter fused silica capillary. The ESI was operated with a spray voltage was 3 kV, nitrogen sheath gas was set to a flow rate of 10 (arbitrary units) with a capillary temperature of 320 °C. The mass spectrometer was operated in negative ionization mode using a previously mentioned adapted method [25]. Acquisition gain control (AGC) target was set to 3 × 10^6^ ions. The samples were analyzed with a resolution of 140,000 and a full scan window of 85 to 800 *m*/*z* for from 0 to 9 min and 110 to 1000 *m*/*z* from 9 to 25 min. Solvent A consisted of 97:3 (*v*/*v*) water:methanol, 10 mM tributylamine, and 15 mM acetic acid. Solvent B was 100% methanol. The gradient was as follows: t = 0 min 100%A 0%B; t = 5 min 80%A 20%B; t = 13 min 45%A 55%B; t = 15.5 min 5%A 95%B; t = 19 min 100%A 0%B; t = 25 min 100%A 0%B with a flow rate of 200 µL/min. Only one analysis mode was used to avoid unknown compounds appearing at different retention times in multiple methods, which would potentially lead to overcounting of replicate compounds.

In broad screening-style untargeted runs, MS^2^ coverage is inherently limited. On tandem mass specs operating in data-dependent acquisition (DDA) mode, only the top 3–15 most intense ions in each duty cycle are selected for fragmentation. Because thousands of MS^1^ features are typically detected in a single injection, selecting only a handful of precursors means that most features never receive MS^2^ spectra. In a recent evaluation of reproducible untargeted workflows, even after de-redundancy only ~15–20% of features were shortlisted as unique precursors, and multiple injections were required to improve coverage [26]. In soil organic matter analysis, standard LC–MS/MS workflows typically yield successful library matches for only ~5% of features, largely due to the limited availability of reference spectra for environmental compounds [27]. Higher annotation rates, around 30%, have been demonstrated using advanced workflows that integrate FT-ICR-MS molecular formula assignments and molecular networking, but these approaches require specialized instrumentation and are not part of typical screening setups [27]. Broader guidance for environmental metabolomics has noted that analyte concentrations in environmental samples are often too low to support comprehensive MSⁿ acquisition, and that most compound identifications rely on a small fraction of the available MS^2^ data [28]. Together, these findings underscore the use of high-resolution MS^1^ detection in this study as the most effective approach for comprehensive coverage and reproducibility in complex soil metabolomics.

### 2.3. Known Spectral Features Processing

Raw spectral files generated by Thermo Scientific Xcalibur [V 4.5.474] software, were converted to the open source mzML format by utilizing the msConvert package from ProteoWizard [29]. After conversion, the mzML files were uploaded to Metabolomic Analysis and Visualization Engine (MAVEN) [30,31]. This program was used for peak alignment and retention time correction. Metabolites were manually integrated by exact mass (±5 ppm) and retention times (Δ ≤ 1.5 min), which were compared to an in-house library of 279 metabolite standards. Instrument drift and batch correction software were not implemented as the small number of samples (<30), did not meet minimum sample set size required of typical normalization methods [32,33].

Statistical analyses were performed in MetaboAnalyst 6.0 [34,35]. The data were normalized by mass, filtered by interquartile range (IQR), log-transformed, and Pareto scaled [36]. MetaboAnalyst 6.0 was used to perform partial least squares discriminant analysis (PLS-DA) for both known and unknown spectral data. From the PLS-DA model, variable importance in projection (VIP) scores were calculated for each metabolite to determine the importance of each individual metabolite for the separation between the groups in the PLS-DA. A VIP score threshold of >1 was used to indicate a metabolite contributed significantly to the different metabolic profiles between groups.

### 2.4. Unidentified Spectral Features

Raw spectral files were converted to mzML format using the msConvert package from ProteoWizard and then each mzML was zipped individually [29]. Zipped mzML were uploaded to MetaboAnalyst 6.0 [34,35]. Within the Spectra Processing tab, the parameters were as follows. UPLC-Orbitrap was selected for the platform. Peak picking used the centWave-auto algorithm with a minFraction of 0.8 for peak alignment in negative mode. Also, contaminant removal was allowed from the chromatogram as well as blank subtraction. The data was deisotoped and deadducted and input into a csv for further statistical analysis.

For the unknown spectral features, intensities were quantile-normalized, filtered by interquartile range (IQR), log-transformed, and Pareto-scaled prior to statistical analyses [36,37]. PLS-DA was used to examine how metabolic profiles differ across the various land management practices. The UpSet plot was created using a free data visualization tool, ChiPlot [38].

Molecular formulas were assigned to the unknown features from MetaboAnalyst using the Seven Golden Rules (7GR) software v46. Only formulas containing biologically abundant elements such as carbon (C), hydrogen (H), nitrogen (N), oxygen (O), phosphorus (P), and sulfur (S) were permitted, to minimize chemically implausible combinations. Mass accuracy constraints were set at 2 ppm for features with *m*/*z* values below 500 Da and 5 ppm for features above 500 Da, in line with typical Orbitrap performance in untargeted workflows [34,35,39]. To enable 7GR’s isotope scoring filter in the absence of curated isotopic data, placeholder values of 5% were entered for A+1, A+2, and A+3 isotopic abundances, with an isotopic abundance error of 5%. This conservative placeholder allows isotope scoring when measured isotopic abundances are unavailable and is appropriate for comparative stoichiometric analyses [40]. An additional filtering step retained only formulas with an isotope match score ≥ 30% to exclude low-confidence assignments [41]. The isotope match score reflects the similarity between observed and theoretical isotopic patterns (relative A+1–A+3 abundances and peak positions). A 30% cutoff was chosen as a conservative compromise that accommodates lower scores introduced by placeholder isotope inputs and measurement noise, yet removes formulas with very poor isotopic agreement. Internally, 7GR applies multiple heuristic rules to improve chemical plausibility, including checks for element ratio ranges (e.g., H/C, N/C), ring double bond equivalents (RDBE), element probability scoring, and isotope pattern matching [39]. These constraints help eliminate implausible or rare combinations while maintaining flexibility for unknown but reasonable organic structures.

When multiple formula assignments were returned for a single *m*/*z* feature (up to three), elemental ratios such as C/N, C/P, and N/P were calculated using a simple average. These averaged formulas are referred to as composite formulas, distinguishing them from individual candidate formulas. This method incorporated structural uncertainty into stoichiometric comparisons by treating all top-ranked formulas equally, rather than assuming a single correct structure. The resulting composite formulas were exported to CSV and analyzed in RStudio v1.3.1093 using a custom R script to calculate elemental ratios and generate violin plots [42,43]. Statistical analysis of stoichiometric ratios was performed using Kruskal–Wallis tests followed by pairwise comparisons against the pasture group only. Effect sizes were calculated to evaluate the magnitude of observed differences.

The same CSV file was processed through a custom Python 3.9 script using the PubChemPy package to retrieve SMILES strings [44,45,46]. Because the focus of this study is on relative elemental composition rather than exact metabolite identification, this approach provides a consistent and chemically sound basis for comparing unknown spectral features across groups. SMILES strings generated from the composite formulas were submitted to NP Classifier (V 1.5), a deep neural network-based tool designed for automated structural classification of natural products [47]. NP Classifier categorizes molecules according to a biosynthetic classification system, assigning each one to hierarchical groups based on biosynthetic class, superclass, and chemical class using learned structural patterns and curated rules.

In practice, each SMILES string was submitted to NP Classifier using a custom Python script that accessed the chemical query interface. Compounds were classified based on key structural features such as alkyl chains, heterocycles, or aromatic rings. This allowed the compounds to be assigned to classes like “fatty acids and conjugates” or specific alkaloid subclasses. This classification approach is based on how compounds are made in nature, making it more relevant for understanding plant and microbial metabolites than general chemical categories. Classification results were exported into a CSV file for downstream analysis, enabling visualization and comparison of chemical class distributions across land management practices.

### 2.5. Quality Assurance and Quality Control

Quality control procedures were implemented in accordance with the Metabolomics Standards Initiative (MSI) Chemical Analysis Working Group guidelines to ensure analytical stability and reproducibility [48]. Two internal standards, L-phenylalanine and L-tryptophan, were added at a constant concentration to all samples prior to extraction and LC–MS analysis to monitor extraction efficiency and instrument performance. Peak areas for both internal standards were integrated following data processing, and their coefficient of variation (CV) was calculated across all biological samples. L-Phenylalanine and L-tryptophan exhibited CV values of 2.28% and 5.78%, respectively, confirming analytical reproducibility throughout the run. Internal standard signal plotted against injection order demonstrated no systematic drift, indicating stable analytical performance across the 24 sample run.

Water blanks were included to assess background signal and potential carryover, and they were also used to determine acceptance criteria for biogenic features. Specifically, only features with peak intensities at least >3× greater than the corresponding signal in the water blanks were retained for downstream analyses, ensuring high confidence that retained features represented true biological metabolites rather than background or non-biological signal [49].

Instrument mass calibration was performed immediately prior to data acquisition following manufacturer recommendations, and mass accuracy for annotated MS^1^ features was maintained within ±5 ppm, consistent with expected Orbitrap performance [50]. Samples were analyzed in randomized injection order to minimize systematic bias. For identified metabolites, intensities were normalized by sample mass prior to log transformation and Pareto scaling [36]. For unknown spectral features, intensities were quantile-normalized, filtered by interquartile range (IQR), log-transformed, and Pareto-scaled prior to statistical analyses, consistent with the workflow described in Section 2.4 [36,37]. Multivariate clustering patterns observed in PLS-DA were consistent with expected biological group separations; therefore, this supports that variance structure was not dominated by run-order or analytical drift. Together, these QA/QC procedures supported data reproducibility and rigor for further analyses.

## 3. Results

An untargeted metabolomics method was employed to investigate the global metabolomes associated with different land management practices. The extraction method and TBA ion-pairing chromatography using negative mode ionization were chosen as they have broad metabolite coverage for extraction and detection, respectively [19,51]. Lipidomics was not performed as water-insoluble compounds are known to be less labile and only present in small quantities in soils [52,53]. This analysis identified 41 metabolites based on exact mass (≤5 ppm) and retention time across all land management soils. Additionally, 4893 unidentified spectral features characterized by retention time and exact mass. These features, considered MSI level 4 compounds, lack structural or class-level annotations at this time [48].

**Figure 1:**
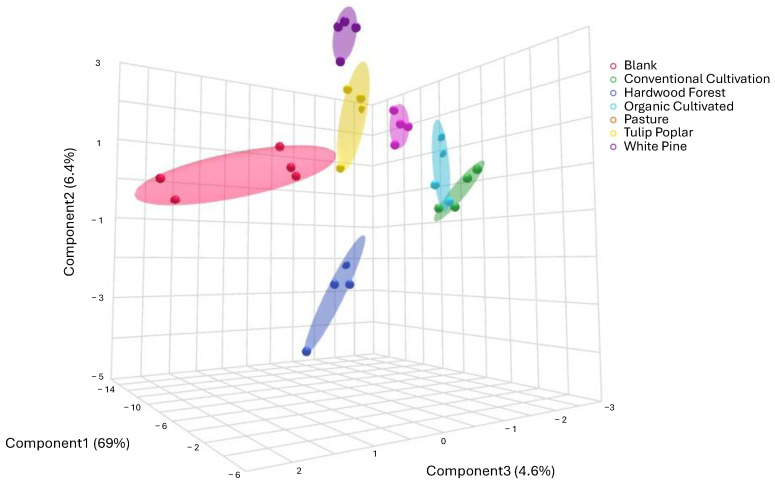
Three-dimensional PLS-DA comparing metabolomes of 41 known metabolites. Identified compounds can be found in Supplementary Figure S6. Experimental groups are shown with 95% confidence intervals.

The 3D PLS-DA using the identified metabolites revealed clear separation among all groups, indicating unique metabolomes for each (Figure 1). A 5-fold cross-validation across 2 components yielded strong model performance (R2 = 0.910, Q2 = 0.814).

**Figure 2:**
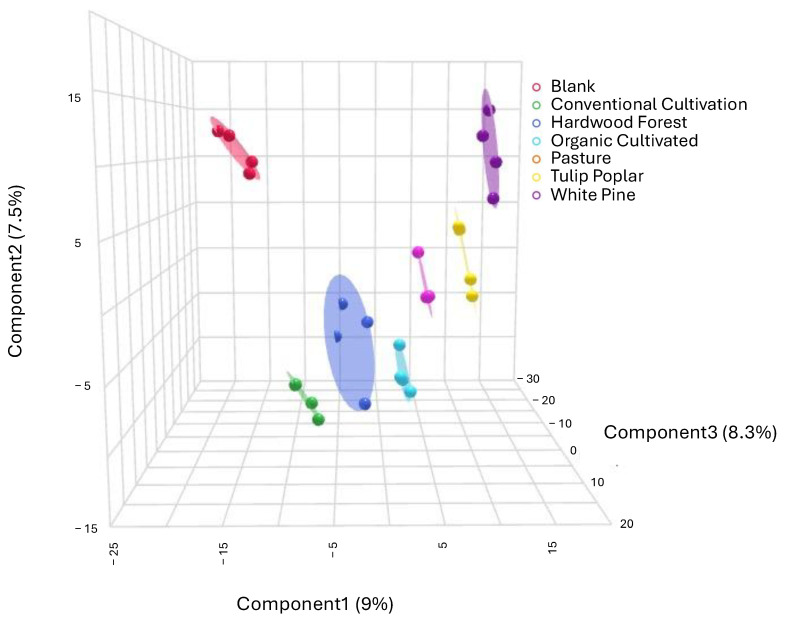
Three-dimensional PLS-DA comparing metabolomes of 4893 unidentified spectral features. Experimental groups are shown with confidence intervals.

Even without structural identification, the 3D PLS-DA using only the unidentified spectral features also achieved robust group separation, highlighting that untargeted spectral data alone can distinguish land management practices (Figure 2). This model, validated using 5-fold cross-validation across 3 components, produced R2 = 0.937 and Q2 = 0.709.

To explore nutrient-related variation among soil metabolomes, elemental ratios were calculated from molecular formulas assigned to unknown spectral features. Ratios included carbon-to-nitrogen (C/N), carbon-to-phosphorus (C/P), and nitrogen-to-phosphorus (N/P). These values provide stoichiometric insight into microbial nutrient availability and organic matter composition. Statistical comparisons were conducted using Kruskal–Wallis tests with pairwise comparisons against the pasture group, and effect sizes were calculated to assess the magnitude of group differences.

**Figure 3:**
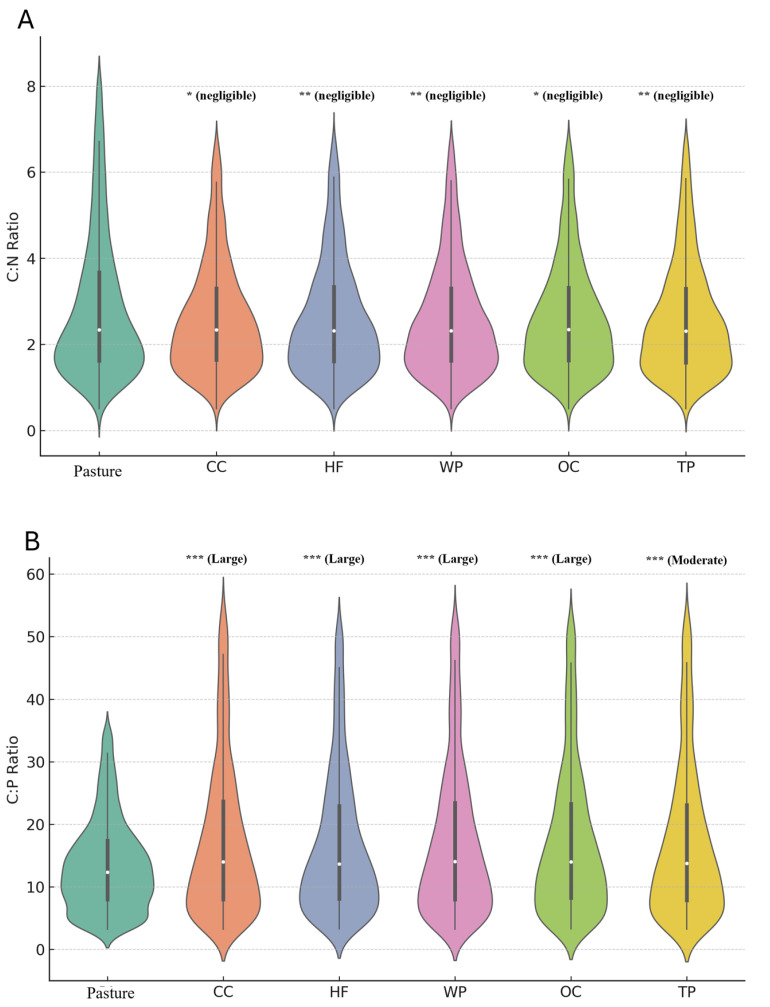

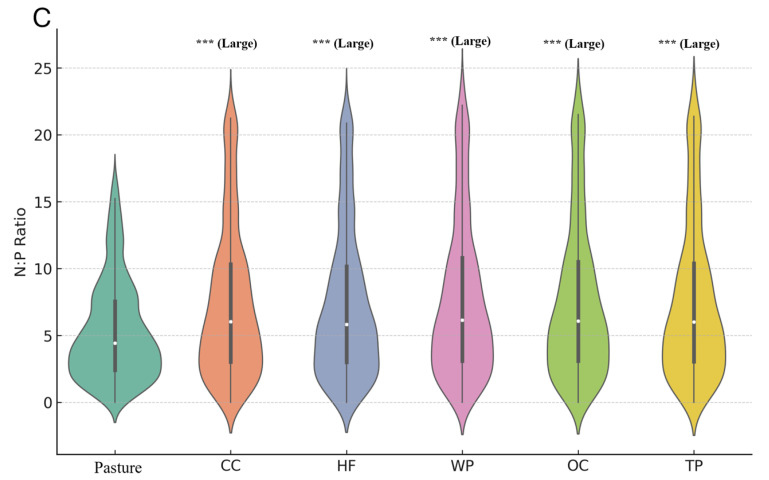
Violin plots show distributions of (**A**) carbon-to-nitrogen (C/N), (**B**) carbon-to-phosphorus (C/P), and (**C**) nitrogen-to-phosphorus (N/P) ratios across six soil types. Statistical significance is based on Kruskal–Wallis tests followed by pairwise comparisons against the pasture group only. Asterisks indicate significance levels (*p* < 0.05, *p* < 0.01, *p* < 0.001), and effect sizes are shown in parentheses. Although some C/N comparisons reached significance, most had negligible effect sizes. In contrast, C/P and N/P ratios showed larger and more meaningful shifts relative compared to pasture.

C/N ratios (Figure 3) exhibited substantial overlap across land use types. Although some comparisons reached statistical significance, nearly all were associated with negligible effect sizes, suggesting minimal ecological relevance. Median values fell between ~2.5 and 3.5, and the interquartile ranges were broad and overlapping. This suggests that C/N ratios derived from labile, water-soluble metabolites are relatively insensitive to differences in land management practices.

In contrast, C/P and N/P ratios (Figure 3B,C) revealed statistically robust separation among groups. Both ratios were consistently lowest in pasture soils and significantly elevated across all other land management types. Pairwise comparisons between pasture and the other five groups yielded large or moderate effect sizes.

The C/P and N/P ratios were also calculated to evaluate whether phosphorus availability may contribute to stoichiometric differences. Kruskal–Wallis tests indicated significant differences in both C/P (*p* < 0.05) and N/P ratios (*p* < 0.05) across land management practices compared to the pasture. C/P and N/P ratios differed significantly across land uses, with white pine consistently exhibiting the lowest values, and hardwood forest and conventional cultivation showing the highest C/P and N/P values, respectively. These findings indicate that, although broad elemental ratios like C/N did not differ significantly may influence phosphorus availability or microbial nutrient acquisition strategies. Violin plots used to visualize these distributions excluded statistical outliers using the IQR method to improve interpretability, though all statistical analyses were performed on the complete dataset. In contrast to the C/N ratio, which showed negligible effect sizes, differences in C/P and N/P ratios exhibited moderate to large effect sizes across land management groups.

**Figure 4:**
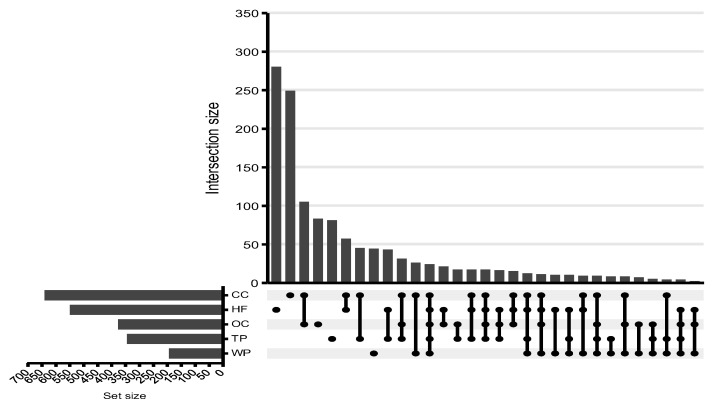
Each land management strategy is represented as a set, and the plot highlights the overlap between them. Each bar’s size represents the number of unidentified spectral features shared between the different combinations of soil types.

To visualize trends in the unidentified spectral data, an UpSet plot was generated (Figure 4). The plot highlights the overlap of statistically significant unidentified spectral features across land management practices, further demonstrating that these unknown features contain biochemically meaningful information.

CC and OC showed significant overlap in features, with 105 shared features between them. Although several groupings share features, most of the sets comprise unique unidentified spectral features. In order of CC, HF, OC, TP, and WP, 248, 276, 90, 55, and 48 unidentified spectral features were unique to their respective land management sets.

**Figure 5:**
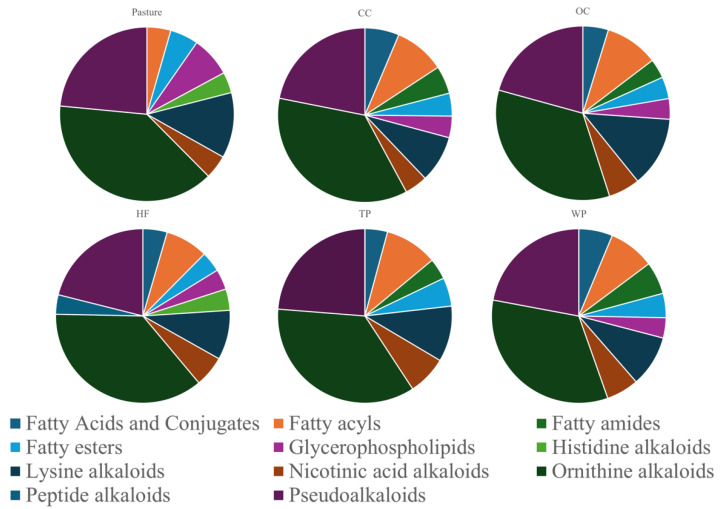
Statistically significant unidentified spectral features, when compared to the pasture, were analyzed using a python script to extract the SMILES Strings from their molecular formulas. These formulas were then categorized by NP Classifier, with the resulting chemical superclasses visualized in pie charts. This approach allowed for an easy comparison of the distribution of chemical superclasses, highlighting the distinct metabolomic profiles associated with each land management practice.

Differences in soil metabolomes resulting from the various land management practices were demonstrated through PLS-DA and PCA. However, to assess differences in elemental composition, the chemical class composition of each soil type was analyzed. Pie charts were created to show chemical classifications from the unidentified spectral features for all soil types (Figure 5). Classification was determined in a cascade from broad kingdoms, superclasses, classes, to more highly refined subclasses.

The individual counts for each category can be found in the Supplementary Table S1. Even without structural information, these data highlight that differentiation between and among soils can be achieved through unidentified spectral features alone. This is a key advantage of untargeted metabolomics in soil science.

## 4. Discussion

This study showed that untargeted metabolomics can distinguish soil systems under different land management practices, even without structural identification of most metabolites. The unidentified spectral features alone were enough to separate land management groups. This means that spectral data can still capture consistent biochemical differences, even when most compounds are unknown.

Only 41 metabolites were confidently identified using an in-house library that focused mostly on human central carbon metabolism. This limitation did not prevent the detection of meaningful differences between land management strategies. While identification helps with interpretation, it was not necessary for group separation. Both identified and unidentified spectral features revealed distinct metabolic profiles across the soil types. Features shared among groups, along with those unique to specific practices, were examined to better understand compositional trends. In addition, elemental ratios were calculated to explore how land management influences nutrient balance and carbon storage. These data provided useful context for how management strategies such as conventional cultivation or hardwood forest affect nutrient cycling.

The distinct metabolic profiles observed across land management types suggest that land use not only changes the availability of carbon and nitrogen, but also shapes the composition of key compound groups. These include superclasses such as fatty acids and conjugates, fatty acyls, glycerophospholipids, peptide alkaloids, and pseudoalkaloids which were all derived through NPClassifier structural taxonomy [47]. The relative proportions of these classes varied by land management type, reflecting shifts in plant-derived inputs, microbial community function, and metabolic processes across soil systems 

To better understand which identified metabolites contributed most to the group separation for Figure 1, we examined variable importance in projection (VIP) scores from the PLS-DA model. The top ten compounds with the highest VIP scores for Component 1 are listed in Supplementary Figure S1. These compounds represent different biological processes related to soil function, including microbial respiration, nitrogen metabolism, sulfur cycling, and organic matter degradation. Also, pairwise comparisons of each experimental group compared to the pasture via PLS-DA and volcano plots can be found in Supplementary Figures S2–S11.

The top metabolite, 2-oxo-4-methylthiobutanoate, is a product of methionine degradation and is involved in microbial sulfur metabolism. It may be more abundant in soils with redox stress or added organic matter [54]. Uracil, a base from RNA, comes from the breakdown of microbial biomass. Its presence suggests high microbial activity and nucleic acid turnover [55]. Glutamine is a nitrogen-rich compound used by microbes and plants to store or move nitrogen. It may reflect nitrogen availability or microbial uptake strategies [56].

UDP-D-glucose is a sugar used to build structural carbohydrates in microbes and plants. It may come from root exudates or microbial polysaccharide production [57]. Pyroglutamic acid is linked to oxidative stress and the glutathione cycle. Its presence may reflect shifts in microbial redox environments [58]. Benzoate is a degradation product of lignin and other aromatic compounds. It often appears in forest soils with large plant litter inputs [59].

Three central carbon intermediates succinic acid, fumarate, and malate are part of the TCA cycle. These compounds reflect microbial energy metabolism and may change depending on oxygen availability or soil organic inputs [60]. Finally, phenylalanine is an aromatic amino acid commonly found in root exudates and plant tissues. It may reflect rhizosphere activity or the breakdown of plant-derived organic matter [61].

Together, these identified metabolites offer a functional perspective on the broader patterns observed in the untargeted data. Even though most compounds were not identified, these few known features highlight processes like microbial respiration, stress response, and carbon and nitrogen cycling. This supports the conclusion that land management affects soil metabolic profiles in ecologically meaningful ways.

### 4.1. Metabolic Profiles Are Distinct Across Land Management Practices

Several common features among groups were observed in the intersections in the UpSet plot (Figure 4). The largest overlap of unidentified spectral features (105) was seen between OC and CC, reflecting their similar agricultural inputs and management strategies. However, each group still maintained distinct biogeochemical profiles shaped by specific land management practices. Despite some shared features, each land management group maintained a largely distinct biochemical profile, emphasizing the influence of management history on soil chemistry. These findings emphasize that soil biogeochemical diversity is closely tied to ecological context and land use, with important implications for soil function and sustainability [62].

### 4.2. Implications of Elemental Ratios

Elemental ratios are commonly used to infer microbial nutrient limitations, organic matter turnover, and biogeochemical cycling in soils. In this study, these ratios were derived from molecular formulas assigned to unknown spectral features, offering stoichiometric insights even in the absence of compound identification.

To visualize broader stoichiometric trends, violin plots of carbon-to-nitrogen (C/N), carbon-to-phosphorus (C/P), and nitrogen-to-phosphorus (N/P) ratios were generated.

To better interpret the practical relevance of these differences, effect size was used to quantify the magnitude of group separation. Unlike *p*-values, which only indicate whether an effect is likely due to chance, effect size describes how substantially groups differ, independent of sample size. Several comparisons in this analysis yielded medium to large effect sizes (*d* > 0.5), indicating that the observed stoichiometric shifts are not only detectable but also potentially meaningful in a biological or ecological context.

This distinction is especially important in metabolomics, where large datasets can yield statistically significant results that lack real-world relevance. Effect size provides a more informative measure of group separation by capturing the strength of the relationship, not just its likelihood [63]. By emphasizing effect size, this study avoids overinterpreting minor differences and instead highlights stoichiometric changes that may reflect meaningful variation in soil metabolite composition.

The C/N violin plot showed only limited ability to distinguish land management practices from the pasture reference group. Although some pairwise comparisons against pasture reached statistical significance, the effect sizes were negligible, and distributions overlapped heavily. These findings suggest that C/N ratios derived from labile, water-soluble metabolites are relatively insensitive to land use driven variation [64]. This is consistent with the interpretation that C/N from metabolomics reflects short-term, bioavailable nutrient pools rather than the broader carbon–nitrogen balance captured in bulk whole soil measurements [65,66].

In contrast, phosphorus-related stoichiometric ratios (C/P and N/P) revealed clearer and more ecologically meaningful differences between land management types. Violin plots showed that cultivated soils, both conventional and organic, had the highest ratios. This pattern may reflect limited microbial access to phosphorus, higher nutrient demand, or leftover effects from past fertilizer use [67]. The distributions were tight and shifted upward, showing that most samples followed this trend. These results suggest that phosphorus availability is more strongly affected by cultivation than nitrogen.

Forested soils also showed elevated C/P and N/P ratios compared to pasture, though not as high as in cultivated soils. White pine soils had slightly lower ratios than hardwood soils. These patterns may be linked to differences in litter quality, decomposition rates, and microbial nutrient use [68]. Forest litter often contains more lignin and complex compounds, which slow phosphorus release and make it harder for microbes to access. In white pine systems, cooler temperatures, lower pH, and slower microbial activity may further reduce phosphorus cycling [69]. In contrast, pasture soils had the lowest C/P and N/P ratios and narrow distributions, suggesting stable phosphorus pools and more efficient phosphorus turnover [67,68]. This may be due to consistent root inputs, low disturbance, and microbial communities adapted to recycling available nutrients. Together, these findings show that phosphorus-centered stoichiometry can reveal how land management shapes soil nutrient balance through both chemical inputs and biological processes.

Even without structural identification, untargeted elemental ratios captured meaningful differences in soil chemistry and inferred nutrient cycling [70]. The strong effect sizes and consistent trends in C/P and N/P suggest that phosphorus availability and microbial nutrient demand vary significantly with land use, and that these differences are reflected in the composition of water-soluble metabolites.

Overall, elemental ratios provide helpful background context, but their ability to detect changes by land use effects may depend on which elements are considered. Future research that combines stoichiometric data with microbial or biochemical markers may better explain how land management influences nutrient cycling [71].

### 4.3. Unidentified Spectral Features Classification

Soil metabolite composition differed by land use, but some patterns were shared across all systems. Certain chemical classes were widespread, while others were restricted to specific land uses. These differences likely reflect the effects of land management on decomposition, microbial activity, and nutrient cycling [72].

Across all land-use types, lysine alkaloids, ornithine alkaloids, and pseudoalkaloids were consistently present in moderate to high amounts. These compounds are commonly produced by both plants and microbes and may reflect universal biochemical processes like nitrogen recycling and stress responses in soil ecosystems [73]. For example, lysine and ornithine derived alkaloids are often associated with plant defense and microbial signaling in the rhizosphere [74]. Their widespread presence suggests they are stable features of soil chemical profiles, regardless of vegetation or management [54].

Free lipid-like compounds (fatty acids and conjugates, fatty acyls, fatty amides, and fatty esters) showed strong variation across land uses. All land-use types except pasture had notable levels of these compounds. For example, conventional cultivation soil had over 25 features in the fatty acid class, while pasture had none. This could reflect rapid lipid turnover in pasture soils, where microbes actively degrade labile lipids from plant roots or manure inputs [75]. In contrast, forest and pine soils may accumulate more of these compounds due to slower decomposition of leaf waxes and other hydrophobic residues [76].

One lipid class, glycerophospholipids, showed the opposite trend. Pasture soils had the highest abundance of glycerophospholipids among all sites. These are structural components of microbial membranes and are often used as indicators of microbial biomass [74]. Their enrichment in pasture suggests a highly active and well-developed microbial community, most likely due to minimal disturbance and continuous plant cover [77]. In contrast, soils that are regularly tilled or used to grow only one crop may have lower microbial diversity and fewer microbial membrane lipids [78].

Nitrogen-rich metabolite classes dominated the soil profiles overall, but some subclasses were exclusive to undisturbed systems. For example, histidine alkaloids were detected only in pasture and hardwood forest soils. These compounds are sometimes produced by soil fungi, especially in undisturbed or perennial systems [79]. The absence of histidine alkaloids in croplands and conifer forests may reflect the loss of fungal diversity due to tillage or simpler plant communities [62].

Peptide alkaloids were absent from all sites except hardwood forest, where they appeared at low levels. This could be tied to the chemical complexity of deciduous litter or specialized microbial interactions in forest soils [80]. Their low levels in farmland and pasture suggest these compounds are not often made in disturbed or grass-based ecosystems [81].

Overall, pasture soil showed a lack of free lipids, high microbial membrane lipids, and unique alkaloids, indicating a fast-cycling and microbially rich environment [82]. Cultivated soils showed signs of lipid breakdown and lacked some microbial or plant-derived alkaloids, pointing to simplified chemical profiles [83]. Forest soils contained more secondary metabolites like peptide and histidine alkaloids, possibly due to complex litter inputs and fungal contributions [84]. Some classes, like ornithine alkaloids and pseudoalkaloids, were shared across all soils. This could suggest that they play a central role in soil chemical cycling regardless of land use [85]. These metabolite patterns show how land use alters not just the quantity, but the types of biochemicals present in soil. This chemical diversity reflects broader ecological functions, including microbial activity, decomposition pathways, and nutrient retention [86].

### 4.4. Implications and Advantages of Metabolomic Approaches in Soil Science

A central methodological advance of this study lies in MSI Level 4 features, spectral features defined only by accurate mass and retention time, were meaningfully incorporated into biologically meaningful analyses with an acknowledgement of the error inherent in their use [48]. Traditionally, Level 4 compounds are often excluded from interpretation due to their lack of structural annotation. Here, we demonstrate that when systematically included, these features provide reproducible and ecologically relevant patterns that differentiate land management systems. This framework helps expand the usable metabolome beyond the small fraction of identified compounds, ensuring that the “dark metabolome” contributes to ecological insight rather than remaining an untapped data fraction [14].

In addition to chemical classification, elemental ratios were calculated for unknown composite formulas, focusing on C/N, C/P, and N/P. Composite formulas are particularly valuable in soil metabolomics, where complex mixtures and limited database coverage often prevent unique metabolite identification. By averaging across the top candidate formulas for a given feature, composite formulas preserve chemical information while reducing the risk of over interpreting any single structural assignment. This approach allows stoichiometric ratios such as C/N, C/P, and N/P to reflect broader chemical trends, rather than being skewed by uncertainty in individual identifications. As a result, composite formulas provide a more stable basis for comparing soil metabolomes across land management practices, offering insight into nutrient-related dynamics even when complete structural resolution is not possible.

In this study, untargeted metabolomics revealed clear differences in metabolic profiles among land management practices. Traditional elemental ratios like C/N, H/C, and O/C did not show significant variation while phosphorous related ratios showed significant differences. This contrast highlights a key advantage of metabolomics: its ability to detect changes in labile compound composition that bulk compound analyses often miss. While standard methods often reflect more stable, organic matter pools, metabolomic data capture short-term dynamics in nutrient availability and transformation [87]. Importantly, these metabolic distinctions emerged even without full identification of all the individual metabolites. Through the use of composite formulas and elemental ratios, unknown spectral features can be processed in a way that preserves stoichiometric information without fragmentation, enabling the detection of meaningful biochemical patterns that distinguish soil management types.

## 5. Conclusions

This work demonstrates how untargeted metabolomics can detect meaningful chemical differences in soils under different land management systems. Even without full compound identification, molecular formulas enable the calculation of elemental ratios such as C/N, C/P, and N/P, which revealed stoichiometric patterns linked to land use. These ratios reflect nutrient-related traits that vary with management and offer insight into microbial demand, nutrient availability, and organic matter cycling.

Structural classification tools like NPClassifier provided a broader view of chemical composition based on biosynthetic origin. Pasture soils showed signs of fast microbial cycling, low lipid accumulation, and distinct alkaloid signatures. Cultivated soils exhibited simplified chemical profiles, often lacking fungal or microbially derived metabolites. In contrast, forested systems contained more diverse and complex metabolites, likely reflecting leaf litter inputs and slower decomposition.

While many detected spectral features remain unidentified, their consistent patterns across land management practices underscore their value in differentiating soil systems. Although the lack of structural characterization limits direct links to specific biochemical pathways or ecological functions, these unidentified features still provide a powerful framework for analysis.

Beyond compound identification, future research should explore the functional implications of these metabolic differences by integrating soil microbial community analyses, environmental variables, and elemental ratio data. Examining metabolic profiles across multiple seasons and environmental conditions could also provide deeper insights into the stability and resilience of soil chemical profiles over time.

Overall, this study highlights the power of untargeted metabolomics in soil science, particularly the ability of untargeted approaches to detect meaningful differences in soil chemistry without requiring full metabolite identification. Continued advancements in compound annotation and functional interpretation will further strengthen the connection between soil metabolic patterns and ecosystem processes, supporting more informed and sustainable land management practices.

## Data Availability

The original data presented in the study are openly available in the Metabolights Repository under accession number REQ20250911213084.

## Supplementary Materials

The following supporting information can be downloaded at: https://www.mdpi.com/article/10.3390/metabo15120783/s1, Figure S1: VIP scores for the top 15 identified metabolites contributing to group separation among land management practices; Figure S2: PLS-DA scores plot comparing conventional cultivation (CC) and pasture soils based on metabolomic profiles; Figure S3: Volcano plot showing differential metabolites between conventional cultivation (CC) and pasture soils; Figure S4: PLS-DA scores plot comparing hardwood forest (HF) and pasture soils based on metabolomic profiles; Figure S5: Volcano plot showing differential metabolites between hardwood forest (HF) and pasture soils; Figure S6: PLS-DA scores plot comparing organic cultivation (OC) and pasture soils based on metabolomic profiles; Figure S7: Volcano plot showing differential metabolites between organic cultivation (OC) and pasture soils; Figure S8: PLS-DA scores plot comparing pasture and tulip poplar (TP) soils based on metabolomic profiles; Figure S9: Volcano plot showing differential metabolites between pasture and tulip poplar (TP) soils; Figure S10: PLS-DA scores plot comparing pasture and white pine (WP) soils based on metabolomic profiles; Figure S11: Volcano plot showing differential metabolites between pasture and white pine (WP) soils; Table S1: NPClassifier superclass counts and percentages for each land management type. Supplementary File S1 and S2: CSVs files for both identified and unidentified spectral features raw peak areas.
