# Supplementary material for: Untargeted Metabolomics Reveals Distinct Soil Metabolic Profiles Across Land Management Practices
Source: Metabolites. 2025 Dec 4;15(12):783. doi: 10.3390/metabo15120783 (PMC12734947; doi:10.3390/metabo15120783)
Supplement: Supplementary file 1 [file metabolites-15-00783-s001.zip › Supplementary.pdf]

Supplementary Materials

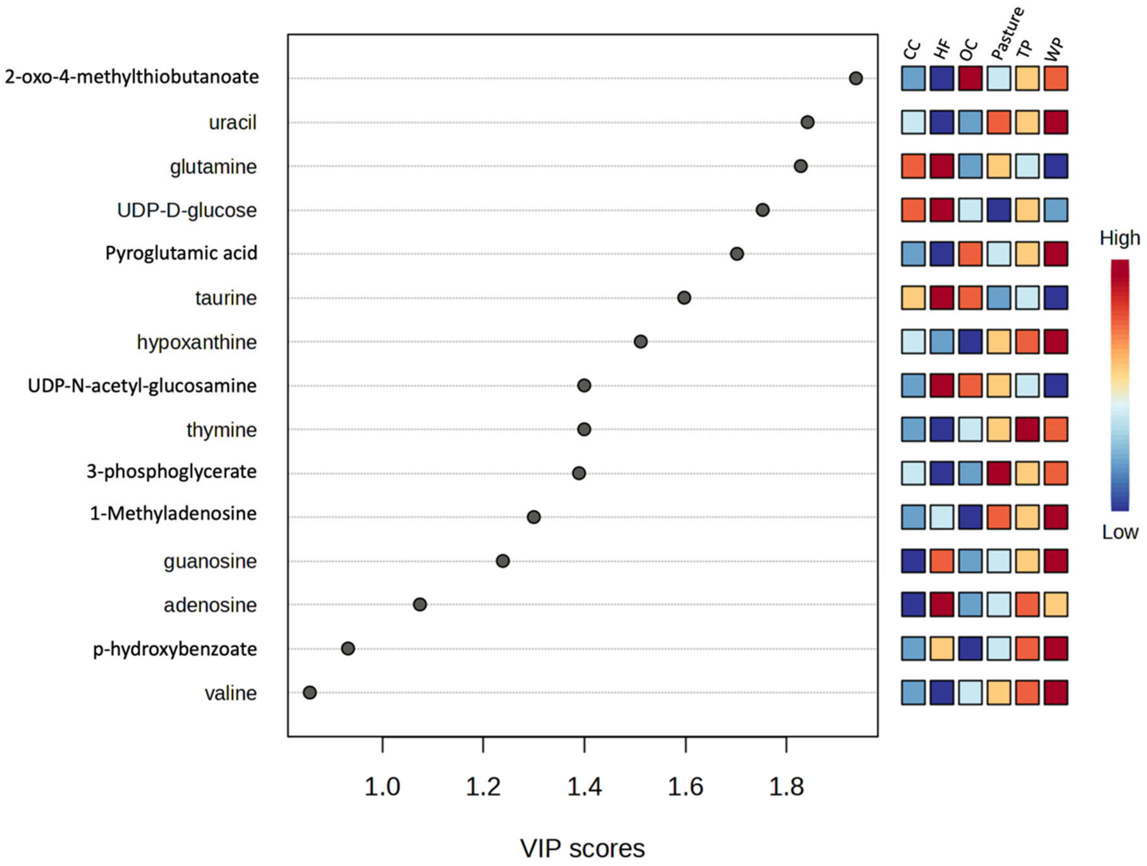

Figure S1. VIP scores for the top 15 identified metabolites contributing to group separation among land management practices. Metabolites were selected based on Component 1 of the partial least squares discriminant analysis (PLS-DA), and all shown compounds had VIP scores greater than 1.0. The accompanying heatmap displays the relative abundance of each metabolite across the six soil groups: conventional cultivation (CC), hardwood forest (HF), organic cultivation (OC), pasture, tulip poplar (TP), and white pine (WP). Warmer colors indicate higher normalized abundance. These metabolites include key intermediates in amino acid metabolism (valine, glutamine), nucleotide turnover (e.g., uracil, thymine), and microbial or plant carbohydrate pathways (UDP-D-glucose, N-acetyl-glucosamine), highlighting distinct biochemical signatures among land use types.

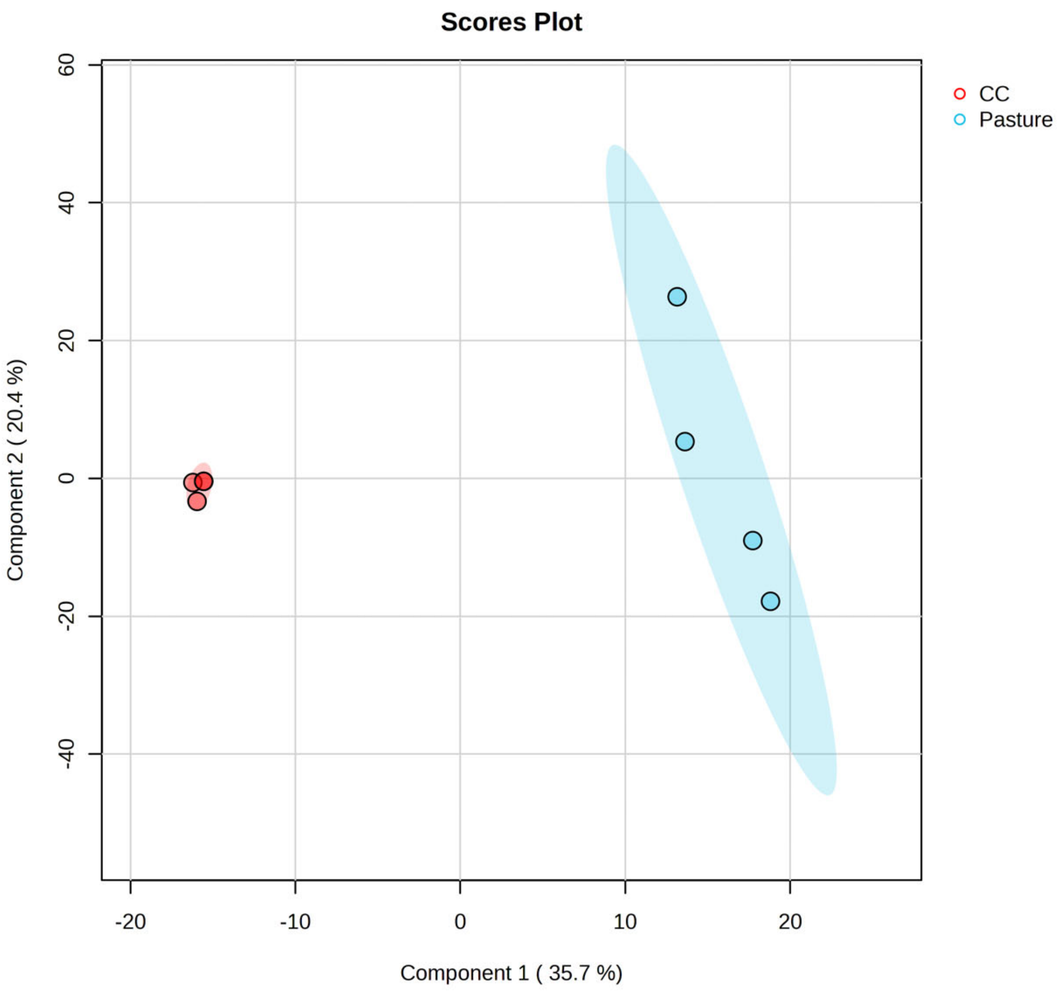

Figure S2. PLS-DA scores plot comparing conventional cultivation (CC) and pasture soils based on metabolomic profiles. The first two components explain 35.7% and 20.4% of the total variance, respectively.

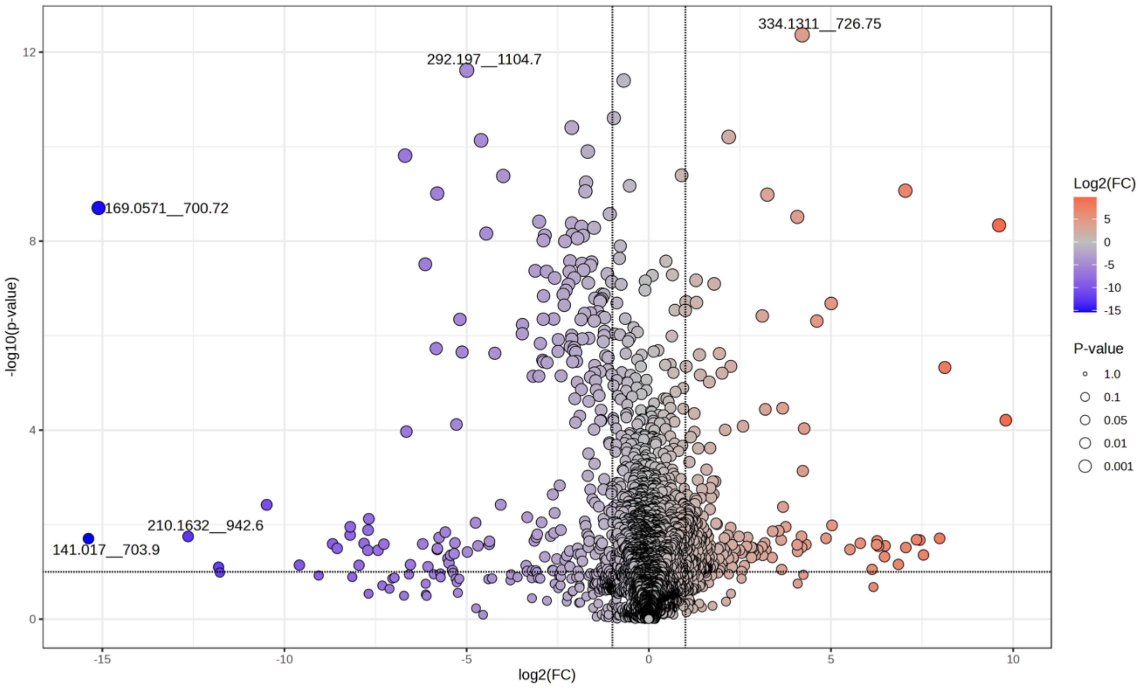

Figure S3. Volcano plot showing differential metabolites between conventional cultivation (CC) and pasture soils. Points represent individual spectral features, with color indicating log_2 fold change and size indicating p-value significance.

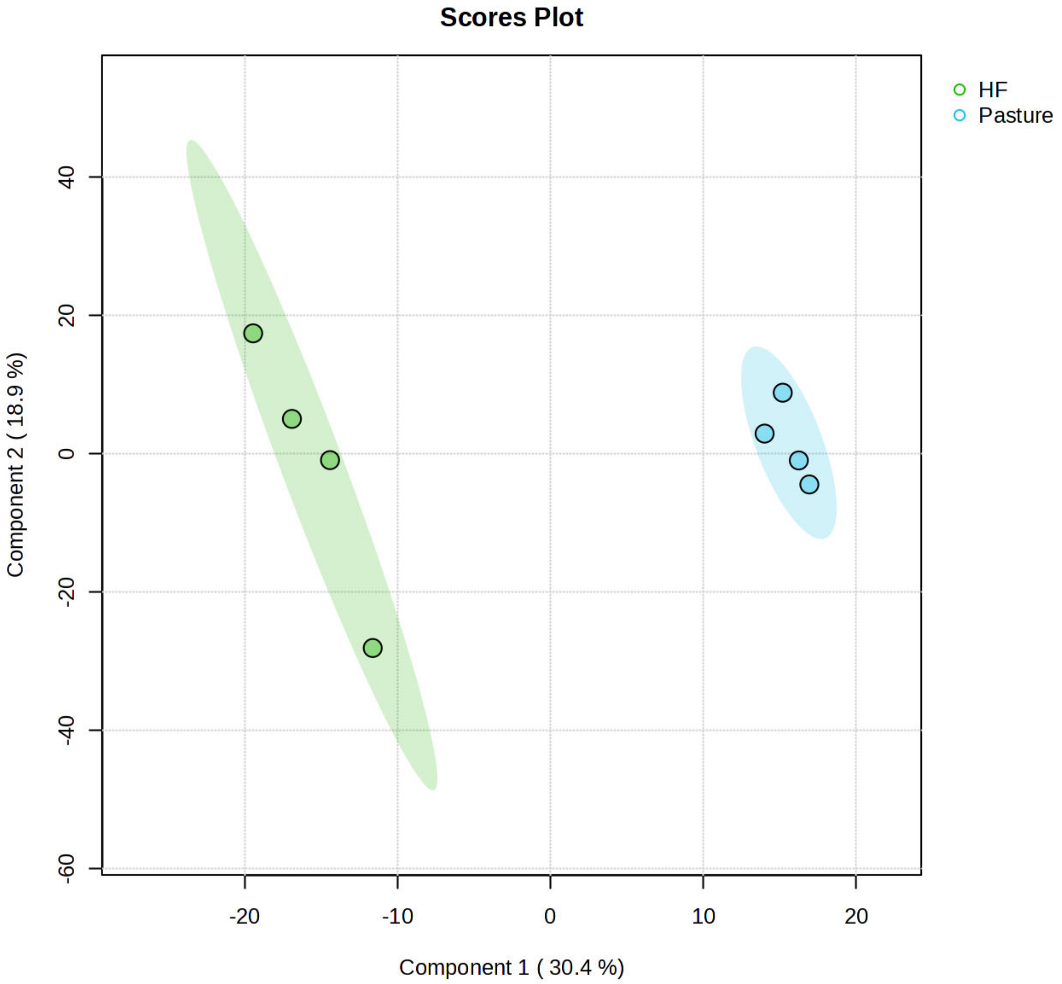

Figure S4. PLS-DA scores plot comparing hardwood forest (HF) and pasture soils based on metabolomic profiles. The first two components explain 30.4% and 18.9% of the total variance, respectively.

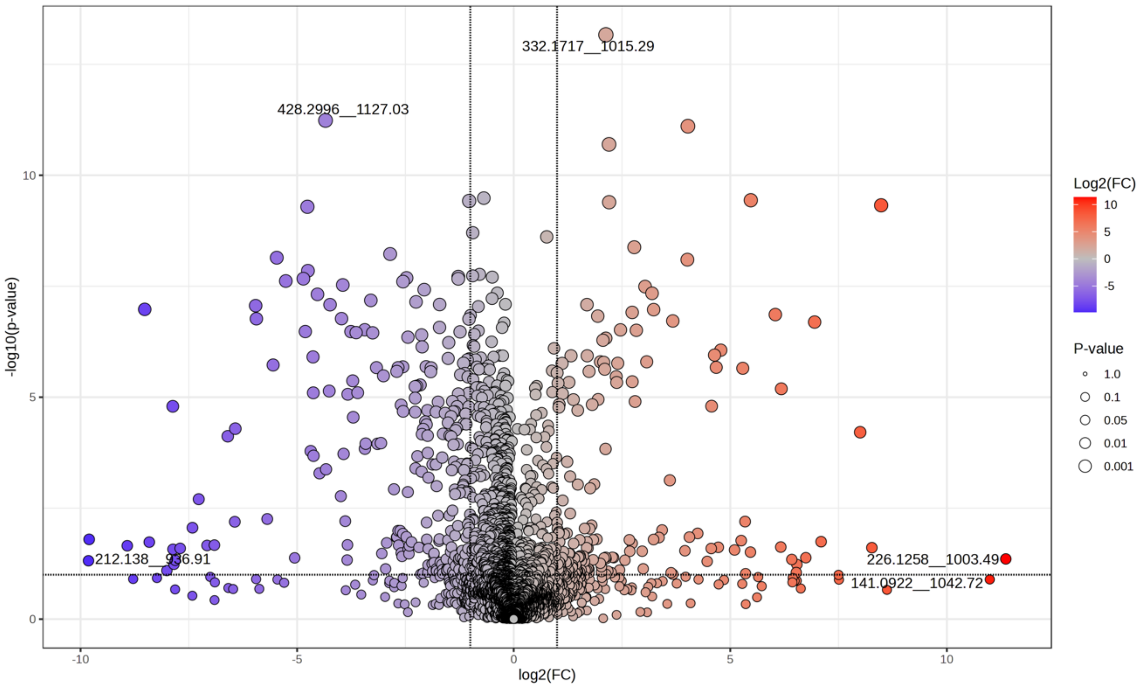

Figure S5. Volcano plot showing differential metabolites between hardwood forest (HF) and pasture soils. Points represent individual spectral features, with color indicating log_2 fold change and size indicating p-value significance.

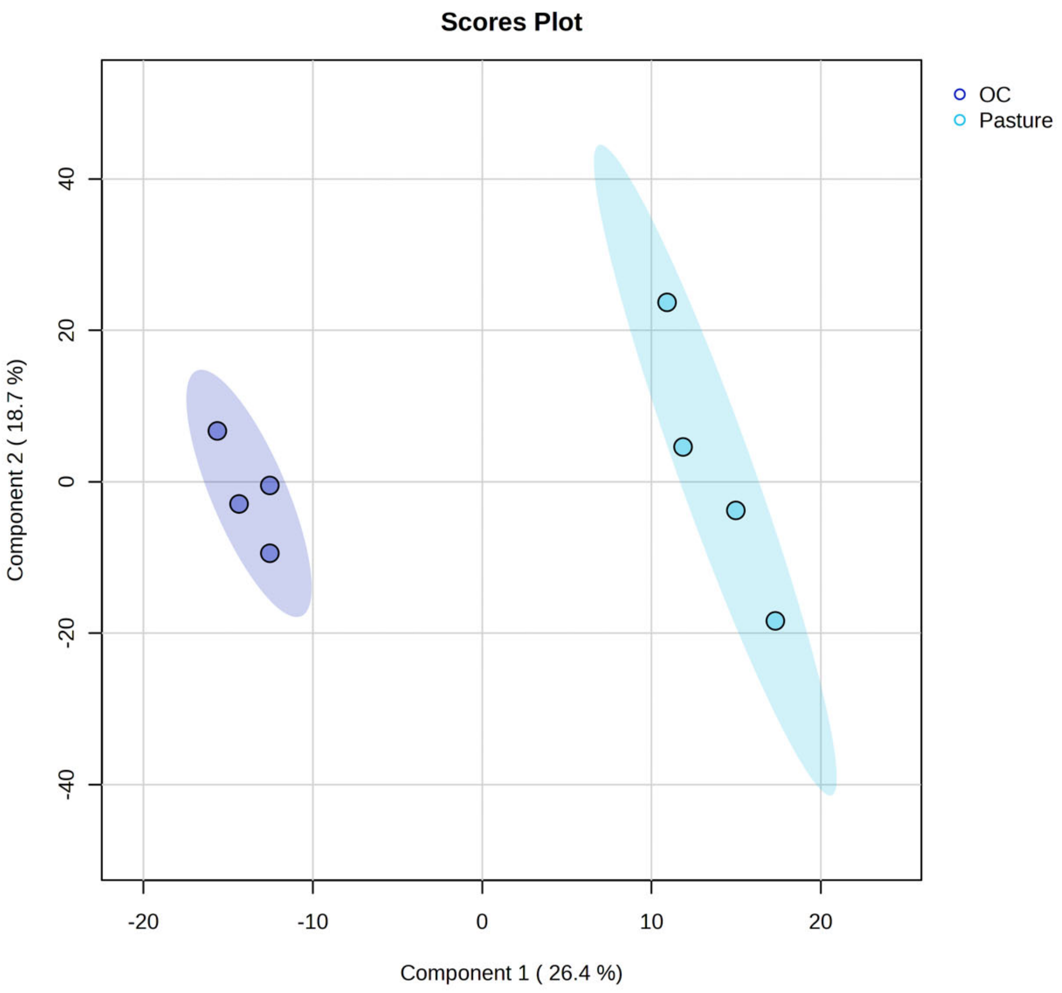

Figure S6. PLS-DA scores plot comparing organic cultivation (OC) and pasture soils based on metabolomic profiles. The first two components explain 26.4% and 18.7% of the total variance, respectively.

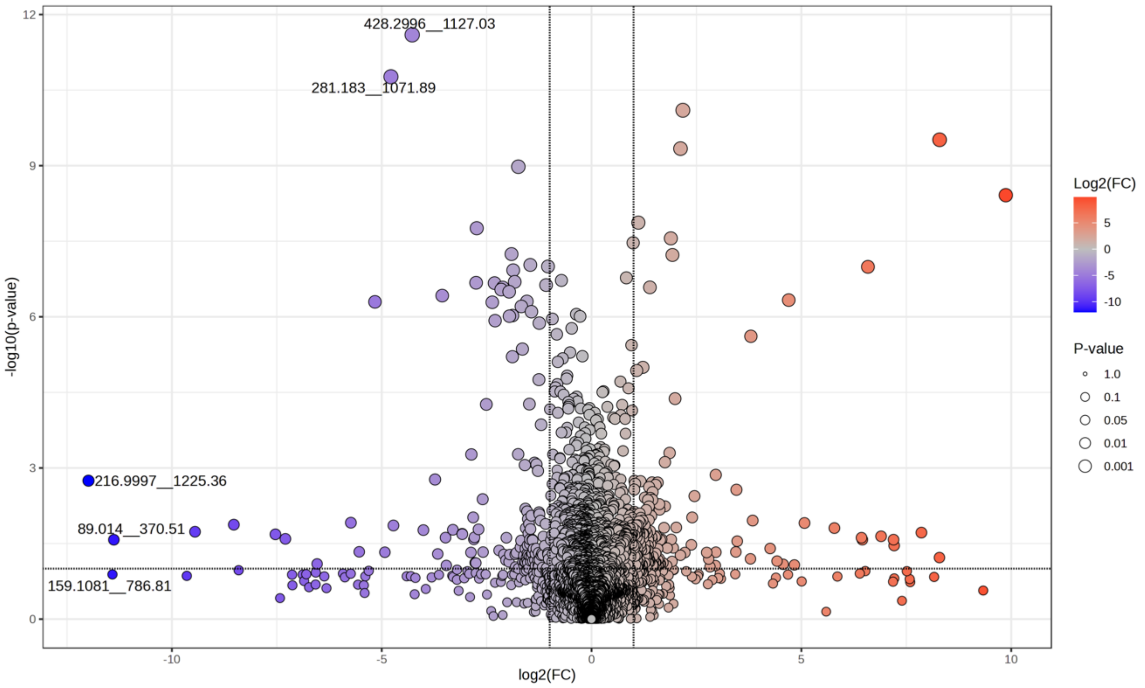

Figure S7. Volcano plot showing differential metabolites between organic cultivation (OC) and pasture soils. Points represent individual spectral features, with color indicating $\log_2$ fold change and size indicating p-value significance.

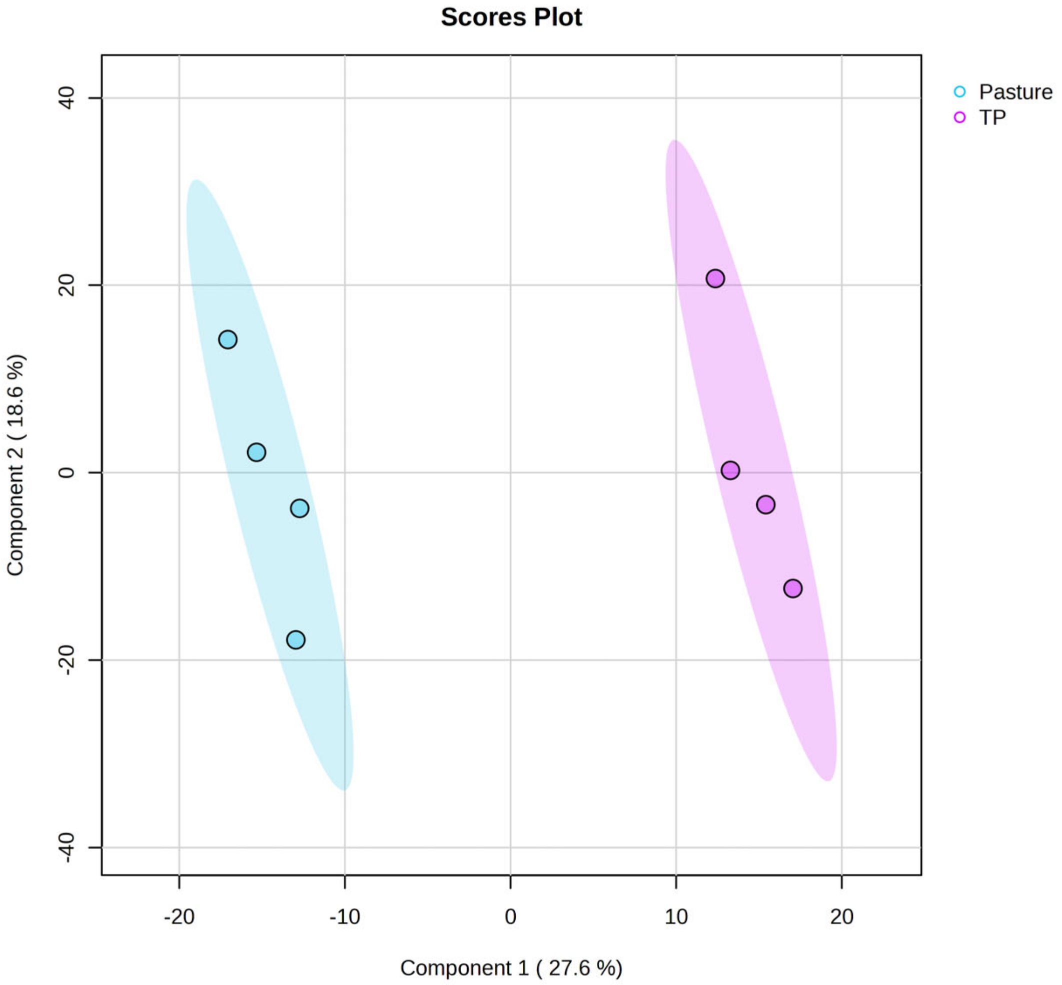

Figure S8. PLS-DA scores plot comparing pasture and tulip poplar (TP) soils based on metabolomic profiles. The first two components explain 27.6% and 18.6% of the total variance, respectively.

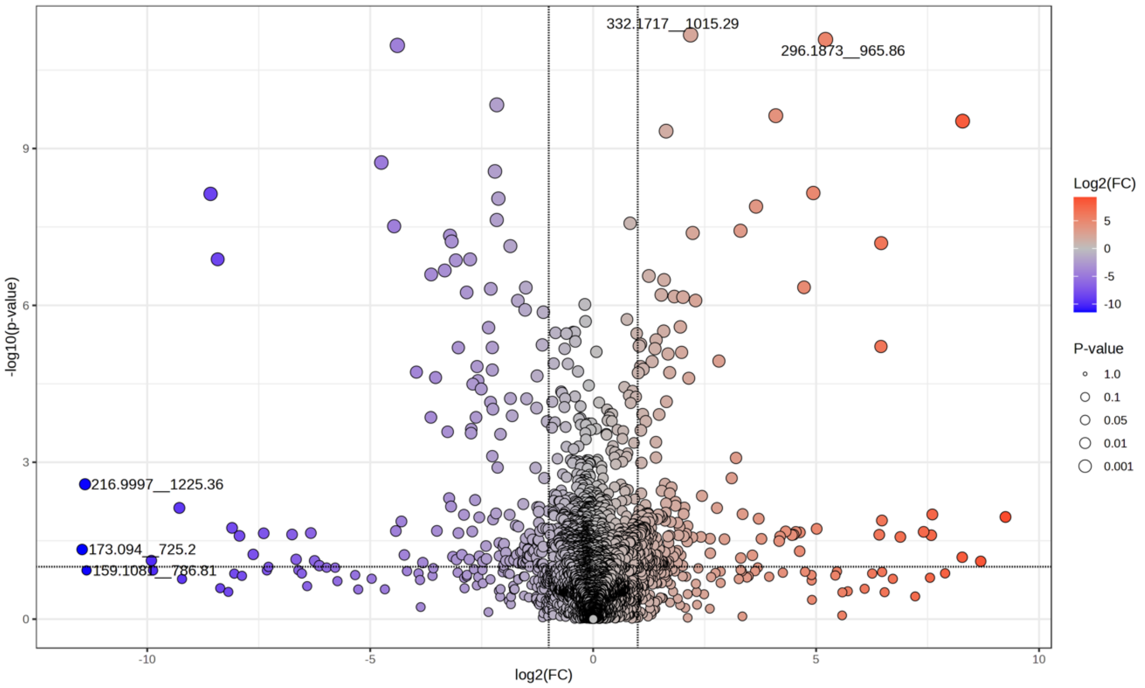

Figure S9. Volcano plot showing differential metabolites between pasture and tulip poplar (TP) soils. Points represent individual spectral features, with color indicating log_2 fold change and size indicating p-value significance.

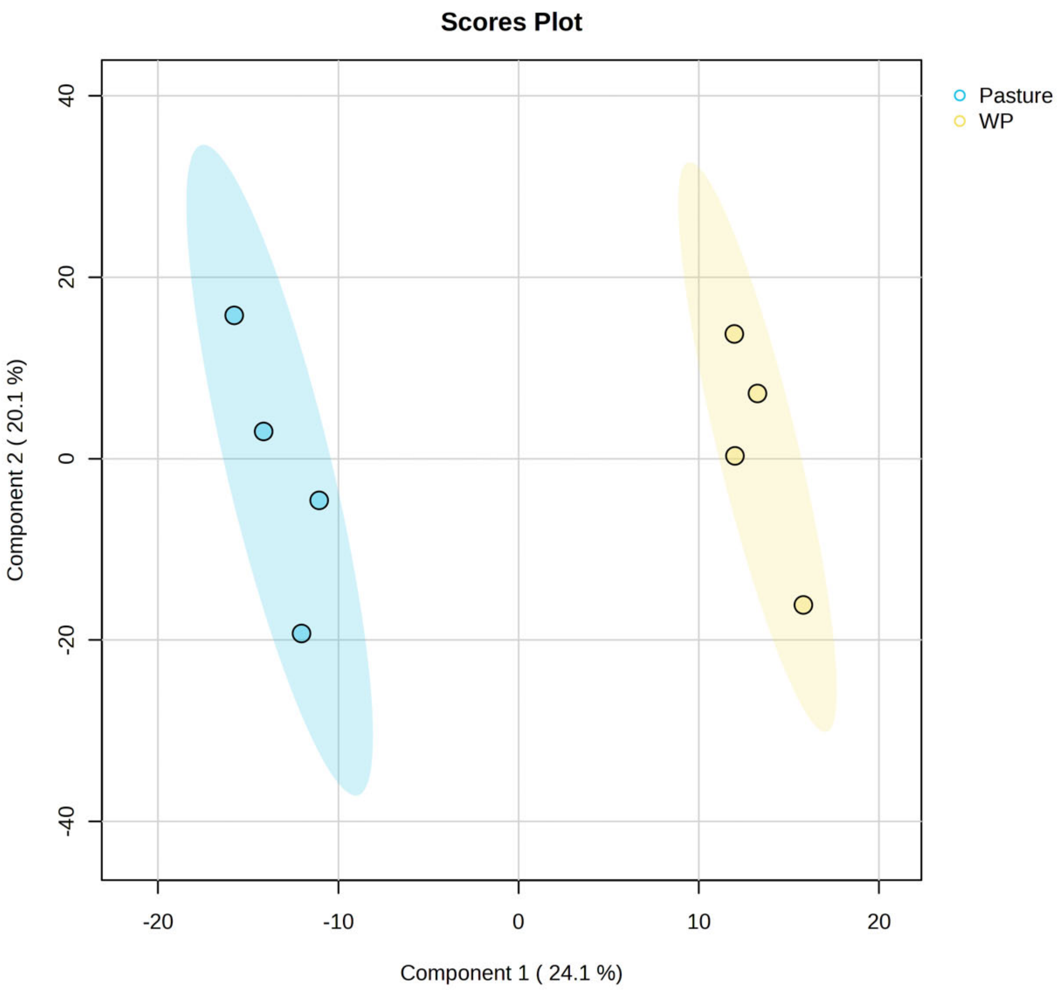

Figure S10. PLS-DA scores plot comparing pasture and white pine (WP) soils based on metabolomic profiles. The first two components explain 24.1% and 20.1% of the total variance, respectively.

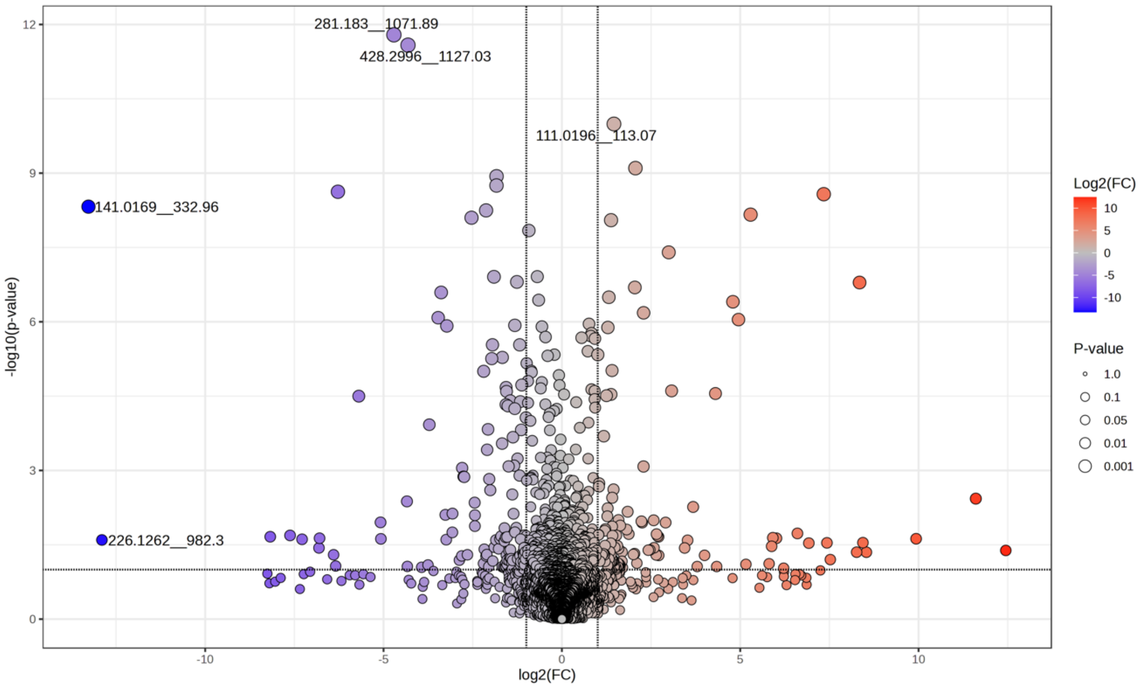

Figure S11. Volcano plot showing differential metabolites between pasture and white pine (WP) soils. Points represent individual spectral features, with color indicating log_2 fold change and size indicating p-value significance.

Table S Error! No text of specified style in document.. NPClassifier superclass counts and percentages for each land management type. Categories representing less than 3% of the total features within a group were combined into the "Other" category prior to visualization in pie charts. Counts are based on classified features only; unclassified features were excluded from both counts and percentages.

**Category**	**HF**	**CC**	**TP**	**OC**	**Pasture**	**WP**
Fatty Acids and Conjugates	25 (3.9%)	27 (5.4%)	15 (3.4%)	21 (4.0%)	0 (0.0%)	25 (5.3%)
Fatty acyls	44 (6.9%)	40 (8.0%)	35 (7.9%)	44 (8.4%)	16 (3.5%)	34 (7.1%)
Fatty amides	0 (0.0%)	22 (4.4%)	14 (3.2%)	16 (3.1%)	0 (0.0%)	24 (5.0%)
Fatty esters	21 (3.3%)	18 (3.6%)	19 (4.3%)	18 (3.4%)	19 (4.1%)	18 (3.8%)
Glycerophospholipids	21 (3.3%)	17 (3.4%)	0 (0.0%)	17 (3.3%)	27 (5.8%)	15 (3.2%)
Histidine alkaloids	22 (3.5%)	0 (0.0%)	0 (0.0%)	0 (0.0%)	14 (3.0%)	0 (0.0%)
Lysine alkaloids	51 (8.0%)	37 (7.4%)	37 (8.4%)	58 (11.1%)	44 (9.5%)	38 (8.0%)
Nicotinic acid alkaloids	32 (5.0%)	18 (3.6%)	26 (5.9%)	26 (5.0%)	16 (3.5%)	24 (5.0%)
Ornithine alkaloids	202 (31.9%)	153 (30.8%)	127 (28.8%)	152 (29.1%)	141 (30.5%)	133 (27.9%)
Peptide alkaloids	20 (3.2%)	0 (0.0%)	0 (0.0%)	0 (0.0%)	0 (0.0%)	0 (0.0%)
Pseudoalkaloids	117 (18.5%)	93 (18.7%)	85 (19.3%)	92 (17.6%)	85 (18.4%)	88 (18.5%)
Other	79 (12.5%)	72 (14.5%)	83 (18.8%)	78 (14.9%)	100 (21.6%)	77 (16.2%)
